# Bridging monitoring gaps in global drylands with big data and collaboration

**DOI:** 10.1038/s44185-026-00129-6

**Published:** 2026-05-16

**Authors:** Tom Bruce, Rajan Amin, Kausik Banerjee, José Carlos Brito, Bogdan Cristescu, Mohammad S. Farhadinia, Matthew Scott Luskin, Brett Lyons, David Olson, Joaquín Vicente, Robert A. Montgomery

**Affiliations:** 1 Zoological Society of London, London, UK; 2 International Big Cat Alliance, New Delhi, India; 3 CIBIO, Centro de Investigação em Biodiversidade e Recursos Genéticos, InBIO Laboratório Associado, Campus de Vairão, Universidade do Porto, Vairão, Portugal; 4 BIOPOLIS Program in Genomics, Biodiversity and Land Planning, CIBIO, Campus de Vairão, Vairão, Portugal; 5 Cheetah Conservation Fund, Otjiwarongo, Namibia; 6 Centre for Environment and Society & School of Applied Sciences, University of Brighton, Brighton, UK; 7 Durrell Institute of Conservation and Ecology, School of Natural Sciences, University of Kent, Kent, UK; 8 School of the Environment, University of Queensland, Brisbane, QLD Australia; 9 The Wildlife Observatory of Australia, QCIF, Brisbane, QLD Australia; 10 NEOM Nature Reserve, NEOM, NC1, Gayal, Tabuk Province, Kingdom of Saudi Arabia; 11 IREC-CSIC-UCLM, Ciudad Real, Spain; 12 Institute of Science and Environment, University of Cumbria, Ambleside, UK

**Keywords:** Conservation biology, Ecology, Biodiversity

## Abstract

Large-scale monitoring networks employing remote sensors have harnessed big data to evaluate conservation efforts on a global scale. While dryland ecosystems are anticipated to expand, and management activities like rewilding and habitat restoration are increasing, the use of data streams and modern analytical methods to plan conservation interventions and quantify their effectiveness remains limited. We recommend establishing a global network for dryland practitioners to bridge critical data gaps, providing two real-world examples. A pilot study in the Kingdom of Saudi Arabia shows how coordinated use of multiple remote sensors and a standardised data pipeline could improve interoperability and facilitate the use of more accurate ecological models. Likewise, the Wildlife Observatory of Australia demonstrates that robust metadata and shared analytical frameworks enable the effective integration of diverse datasets using hierarchical occupancy models. Key steps to build this network include forming a steering committee, engaging stakeholders from various backgrounds, piloting projects in different regions, agreeing on protocols and exploring seed funding opportunities.

## Introduction

### The global proliferation of ‘big data’ and linked sensor networks

Large-scale monitoring networks employing standardised methods of data collection can act as early warning systems for ecosystem change or degradation^1^. Networks of remote sensing technologies, including camera traps, passive acoustic monitors (PAM), GPS telemetry, unmanned aerial vehicles (UAVs) and satellite monitoring, enable scientists to assess a broad range of impacts on wildlife, from climate change to local human activities^2–4^. Existing monitoring networks have predominantly focused on tracking species occurrence, sometimes linking the patterns to drivers of change such as land cover (e.g. Snapshot programmes, European Observatory of Wildlife, Wildlife Observatory of Australia) or in areas of high biodiversity, such as tropical rainforests (sensu^5,6^). Existing networks typically employ a single monitoring method, most often camera traps^7^, especially in biomes with dense vegetation like rainforests, where direct sightings and field signs, such as tracks and scat, are less suited^8^. With rapid advances in technology, there is now a huge opportunity to establish systems and protocols to integrate multiple sensor data streams at scale. By combining multiple data streams, such as camera trap data and satellite imagery, researchers can address questions across varying spatial and temporal scales. Satellite data, for example, has been used to provide information on plant phenology or landscape fragmentation, offering environmental context to explain wildlife patterns observed by camera traps^9^. This integration allows predictive models to operate at scales that extend beyond individual sites and landscapes^10,11^. Such integration and standardisation will provide much-needed information to help optimise conservation practices and adaptive management interventions at the landscape level^12,13^.

There is increasing recognition of the importance of dryland ecosystems (hereafter, referred to as drylands) for ecosystem services, human livelihoods and conserving biodiversity^14–16^. Habitat restoration and rewilding initiatives are becoming more prevalent in drylands^17^. Presently, despite global advances in remote sensing and wildlife monitoring^18–22^, there is a comparative dearth of monitoring programmes in drylands to quantify wildlife population dynamics and habitat associations with environmental and anthropogenic variables post-intervention. To address the current monitoring gap in drylands, we propose a collaborative network of scientists and practitioners, similar to other global initiatives such as the Tropical Ecology Assessment and Monitoring (TEAM) network, be established that would enhance understanding and drive progress in dryland conservation worldwide. Expanding existing networks is unlikely to be effective, as these platforms have seen limited adoption for reasons that remain unclear, potentially including inadequate technological infrastructure, data restrictions imposed by non-disclosure agreements and general reluctance to collaborate. Moreover, dryland practitioners often rely on multiple data sources to account for low species densities and wide-ranging wildlife, which existing frameworks are not designed to accommodate. Finally, the relative paucity of research in dryland systems means that a dedicated, community-informed network would better align with local monitoring needs and promote broader participation. A bespoke network would facilitate standardisation, scaling up and subsequent information sharing to assess changes in these systems at biogeographic scales and help achieve more effective and sustainable conservation outcomes.

### Dryland ecosystems

Drylands are defined as areas with an aridity index of <0.65^23^ and comprise about 42% of the Earth’s land surface^24^. Worst-case projected aridification climate models suggest that by 2100, half of the world’s landmass could be classified as drylands, an area in which 5 billion people currently inhabit^25,26^. Contemporary research shows that drylands play a more substantial role in global biodiversity than previously recognised. For example, Gross et al.^27^ provided empirical and mechanistic evidence to support a ‘dryland functional paradox,’ where plant phenotypic diversity displays an unexpectedly broad range of forms and functions despite strong environmental filtering. This finding highlights the importance of drylands in sustaining global-scale biodiversity, even under harsh conditions. Yet, despite their ecological significance, drylands have historically received limited conservation attention, even at small scales, due to chronic underfunding of biodiversity initiatives^28,29^. Consequently, local capacity and resources to address biodiversity loss and promote community-driven conservation remain scarce^30^. Within drylands, anthropogenic pressures are among the greatest threats to the persistence of biodiversity^31^. These include agricultural and infrastructure expansion, overgrazing and a loss of traditional pastoralism, overexploitation of wood and plants, direct hunting, and human-wildlife conflict^32^. More frequent and extreme weather patterns, driven by global warming, interact synergistically with anthropogenic pressures, further exacerbating species decline and ecosystem degradation^32^.

There is a paucity of baseline information on wildlife communities and ecosystem functions in drylands^33^. Wildlife in drylands often exists at very low densities, which makes long-term monitoring challenging and complicates statistical inferences due to insufficient data. Moreover, their movements are highly unpredictable due to scarce and variable resources, creating significant challenges for sampling, area coverage and linking individual behaviour to population-level patterns^34,35^. In less complex food webs, the loss or introduction of even a single species can have far-reaching and unpredictable implications for the ecosystem^36,37^. Trophic cascades occur when predators influence species diversity and/or abundance at different levels in a food web, either or both directly and indirectly^38,39^. The strength and extent of the cascading mechanisms depend on several factors, including bottom-up versus top-down limitations and the nature of interspecies interactions.

Ecological theory predicts that cascades are more likely in drylands because they are generally bottom-up systems with limited and sporadic patchily distributed resources^40^. While drylands can provide conditions for remarkable species endemism and select for unique adaptations that enable survival in the harsh environment^27^, bottom-up systems also reduce species diversity and increase interaction strength within and between trophic levels^41^. Increasing research into the broader effects on ecosystem structure and function resulting from species losses and reintroductions in dryland systems remains crucial for predicting how these systems will change. This is particularly important as practitioners can aim to reintroduce species or manipulate entire communities by employing trophic cascades to restore ecosystems to previous benchmarks in time.

### How would a global network help?

**Fig. 1:**
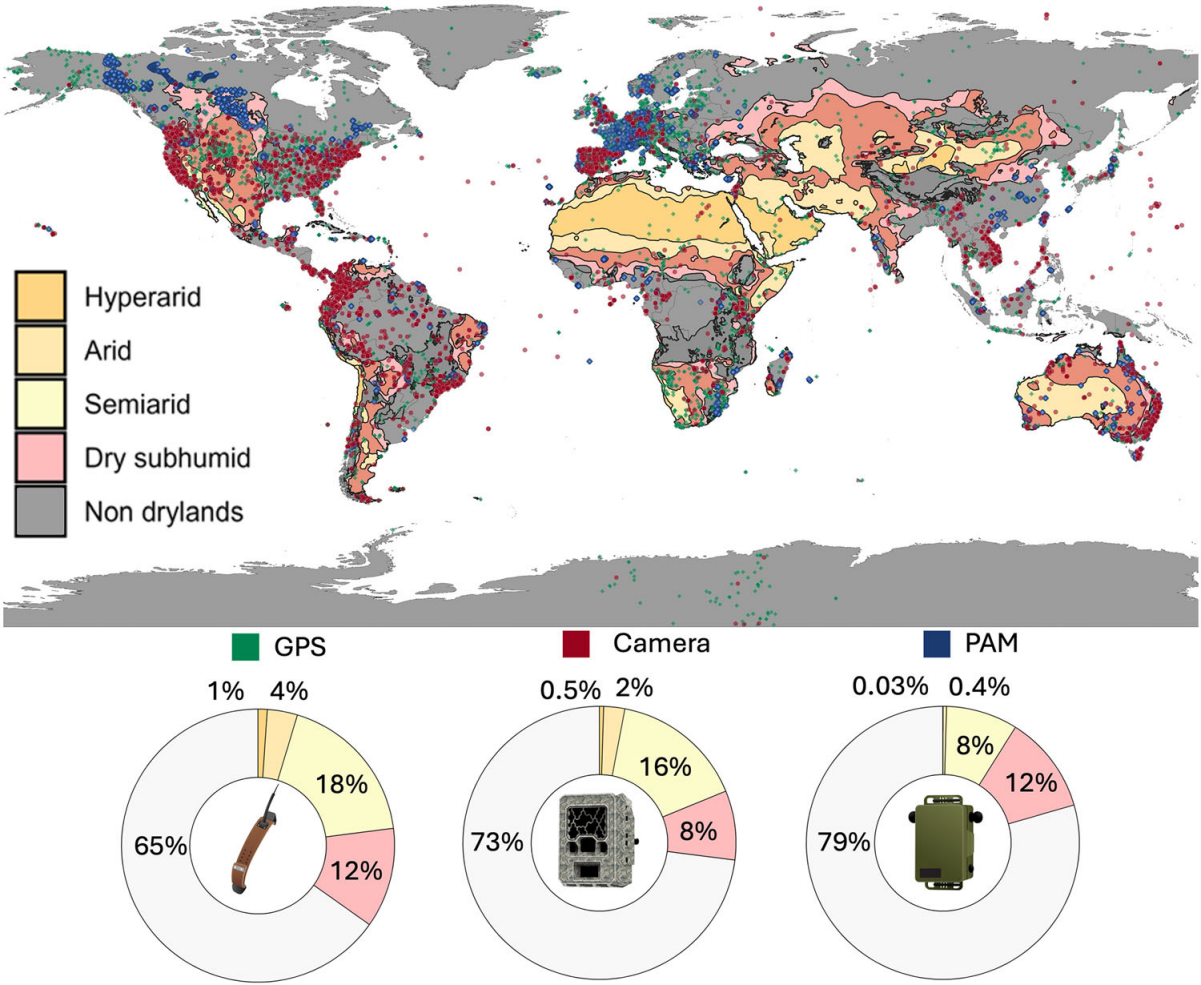
Red points represent camera trap data from Wildlife Insights^107^, blue points represent passive acoustic monitoring (PAM) data from Worldwide Soundscape^19^ and green points represent animal tracking (GPS) data from Movebank^108^. The map highlights spatial gaps in data coverage, particularly in dryland systems outside of the USA and Europe. The doughnut plots below the map show the proportion of monitoring locations occurring within each terrestrial biome, emphasising the underrepresentation of drylands, particularly hyperarid and arid, compared to other ecosystems. Dryland habitat data from: UNEP-WCMC (Dryland Habitat Dataset)^109^.

Other successful ecological monitoring networks have emphasised the creation of a knowledge and information-sharing community^6,42^. Establishing a global network of practitioners in drylands would help alleviate some of the factors that may hinder managers and scientists from adopting automated sensors^43^. Based on datasets contributed to major biodiversity data repositories, dryland systems are markedly underrepresented, with substantial gaps across most regions outside Europe and North America (Fig. 1). Despite hyperarid and arid systems covering approximately 7% and 11% of the Earth’s surface^44^, respectively, together they account for less than 3% of the data archived in each of these repositories. To increase the use of sensor arrays, such a network would need to provide standardised protocols, multilingual support, analytical workflows, and tutorials for various stakeholders, including wildlife managers, governmental and non-governmental organisations, and academics (Table 1). We present an approach that categorises the data-gathering process into ‘sensors’, enabling animal tracking and habitat mapping. We also incorporate ‘direct observation methods’ such as technology-aided ranger patrols (e.g. Earth Ranger, Spatial Monitoring And Reporting Tool (SMART)) or traditional transect and plot surveys.

**Table 1:** Suggested minimum recommended spacing, sampling sites, and duration for sensor-based and direct observation methods suitable for dryland systems, to facilitate key analyses and tools that can be employed for pre-processing and analysis of data

Method	Spacing-strategy	Sampling sites/ individuals	Duration	Analysis methods	Metrics	Rationale	Pre-processing/analysis software	Refs.
Camera traps	>1 km–≤5 km—Systematic grid	>40	>2 months	● Spatial capture-recapture methods ● Distance sampling ● Time-lapse methods ● Single- and multi-species occupancy methods ● Hierarchical diel activity models ● Species richness models ● Rarefaction curves	● Density ● Occupancy ● Species richness ● Relative Abundance Index ● Activity patterns ● Environmental change (phenology)	**Target taxa:** mammals, terrestrial birds and large reptiles. **Environmental assessment:** Phenology, fire events, human activities, e.g. driving, hunting and domestic animals. Cameras should be spaced far enough apart to cover a representative area and be larger than the target species’ home range. This is not critical for any method other than occupancy, and if violated, the output is the probability of site use. At least two months is a reasonable timeframe for occupancy, REM, and distance sampling. Longer durations and greater spacing may be required for cryptic/low-density species. High-frequency time-lapse pointed directly at the ground can be used for small-sized species (small mammals, reptiles, macro-invertebrates) with subsequent application of time to event, and similar models for density. Such an application may require a reduced spacing between cameras.	● AI image classifiers (Megadetector, Deepfaune, Wildbook) ● Zoological Society of London Camera Trap Analysis Package ● Agouti ● CaTRAT (Camera trap data repository and analysis tool) ● R packages (Distance, SpOccupancy, vegan, camtrapR, GLMMadaptive, brms, activity)	^65–82^
Passive acoustic monitors (PAM)	> 1 km–≤5 km—Systematic grid	>40	>2 months	● Single- and multi-species occupancy methods ● Hierarchical diel activity models ● Species richness models ● Rarefaction curves	● Occupancy ● Species richness ● Relative Abundance Index ● Activity patterns	**Target taxa:** vocal species, predominantly birds, amphibians and bats, but some mammals, e.g. wolves are possible. **Environmental assessment:** Anthropogenic noise, e.g. gunshots, chainsaws and vehicles PAM should be spaced far enough apart to cover a representative area and be larger than the target species’ home range. This is not critical for any method other than occupancy, and if violated, the output is the probability of site use. At least two months is reasonable for occupancy. Longer durations may be required for cryptic/low-density species.	● BirdNET analyzer ● RavenPro ● R packages (SpOccupancy, vegan, GLMMadaptive, brms, activity)	^74,76,77,79,82–85^
UAV transects/ Line transects	*Uniform spacing between transects—Perpendicular to environmental gradients	>20	N/A	● Distance sampling ● Object- based Image Analysis (OBIA)	● Density ● Relative Abundance Index ● Vegetation cover	**Target taxa:** Larger mammals visible from height, ground nesting birds. Environmental assessment: Vegetation cover, anthropogenic disturbance, e.g. tyre tracks, land use classification change. This approach needs at least 20 transects to capture the underlying variance in encounter rate. Duration is not essential, but transects should aim to be completed in as few days as possible. Pilot studies make estimating the initial survey effort required more reliable. Normally >30–40 observations provide reasonable estimates.	● Open drone map ● Clickpoints ● R packages (Distance, dsims)	^81,86–92^
GPS telemetry	N/A	NA	?	● Resource selection function ● Step selection function ● Kernel density estimates ● Hierarchical diel activity models	● Habitat preference ● Activity patterns ● Home range	**Target taxa:** predominantly used for larger mammals, but can be used for smaller species, assuming the GPS unit is lightweight enough. Guidelines for the number of individuals required for robust metrics are currently lacking. The nuances of wildlife behaviour in drylands generate significant autocorrelation in GPS telemetry datasets. For instance, the higher levels of exploratory movement observed in rewilded animals post-release, remaining near water for extended periods, and nomadic movements away from resource points all contribute to unique patterns in the data. Path segmentation and path clustering can be used to refine this data source.	● R packages (ExMove, ctmm)	^93–97^
Ranger data collection	N/A	N/A	N/A	● Single- and multi-species occupancy methods ● Rarefaction curves ● Species richness models	● Occupancy ● Species richness ● Relative Abundance Index ● Catch per unit effort ● Differenced plots ● Environmental change (phenology) ● Disturbance processes (e.g. fire, illegal hunting)	Ranger-based data collection is a vital component of monitoring in protected or community-managed areas, as data on wildlife and human activities are collected while patrolling. The main challenge in utilising these data is ensuring that they are both efficient for rangers to gather and can be analysed to provide robust metrics useful for protected area management and early warning.	● Spatial Monitoring and Reporting Tool (SMART) ● EarthRanger ● R packages (SpOccupancy, R2WinBUGS, vegan)	^76,77,98–103^
Satellite remote sensing/ UAVs	N/A	N/A	N/A	● Object based image analysis (OBIA) ● Spectral separability analysis ● Random forest	● Environmental change (phenology) ● Landscape use classification ● Vegetation coverage and quality ● Disturbance processes (e.g. fire, habitat fragmentation)	Satellite remote sensing is an emerging field at the intersection of ecology and big data. Data is gathered at increasingly fine resolutions, down to 1 m from satellites; however, UAVs also permit measurements at finer resolutions, down to millimetre scales if needed. By using remote sensing data, researchers can apply cross-scale approaches to link how changes at small spatial scales relevant to individual plants or animals can affect overall processes and patterns observed at a landscape scale. This becomes more powerful with repeated surveys as environmental patterns and change become observable across variable spatial and temporal scales. UAV and satellite remote sensing measurements can be directly complementary if conducted simultaneously with other monitoring methods, such as camera traps, PAM, or line transect surveys, which can be used as explanatory variables when modelling wildlife population trends or detectability.	● QGIS ● Open Drone Map	^104–106^

To ensure global coverage and engagement, groups representing transboundary initiatives, such as the International Union for Conservation of Nature (IUCN) Commission on Ecosystem Management Dryland Ecosystem Specialist Group, the China Drylands Initiative and Sahara Conservation, as well as research groups in countries with drylands, should be engaged from the outset. An initial consultation phase is essential for representatives to ascertain the key components of the proposed network, including the standardisation of methodologies, the set of metrics to be used for monitoring, and the planning of additional activities, such as in-person training workshops (Table 2).

**Table 2:** Potential training and capacity-building activities throughout the lifetime of a collaborative network

Event type	Components/Topics	Expected output
Networking and meetings	● Consensus on standardised methodology ● Partnerships ● Funding ● Knowledge exchange ● Data sharing agreements ● Collaboration between practitioners across boundaries	● Data sharing protocol ● Standardised data collection protocol ● Partnerships with policy and practice stakeholders
Training and capacity building	● Study/survey design ● Analysis and reporting of data ● Research ethics	● Recorded seminars hosted on the YouTube channel ● Best practice for deploying survey devices ● Code scripts used in workshops ● Summary reports from workshops are distributed to the network
Policy	● Synthesising information for policy ● Presenting at policy events	● Evidence reports and publications ● Policy events supplemented with network findings ● Transparent data sharing agreement capable of being used and promotes a trusting environment: ‘*sharing data is beneficial and often the only way for addressing major issues*’

The chosen metrics and analyses are likely to favour hierarchical modelling approaches, which allow for variation in deployment strategy by accounting for the effects of different factors on the probability of detecting wildlife. Hierarchical models are particularly advantageous because they separate the ecological state process (e.g. abundance or occupancy) from the observation process (detection), allowing uncertainty to be propagated through the analysis and reducing bias in population estimates. This is especially important for large datasets generated by devices such as camera traps and PAMs, where imperfect detection causes missed observations even when animals are present.

Hierarchical models can also account for spatial heterogeneity in both detectability and the ecological state process, which will naturally occur in any large-scale monitoring network. For the chosen monitoring metrics, such as animal density or occupancy, the network should provide example analysis scripts and clearly outline the strengths and limitations of different metrics for its members. Accounting for detection probability enables the deployment strategy to be flexible and evolve as practices and scientific thought change, accommodating unique use cases when required by practitioners. Local ecological knowledge, which has been shown to enhance long-term monitoring programmes, should be encouraged for adoption by members where possible from the outset^45^.

### Socio-economic barriers to big data

In many countries with drylands, several socio-economic conditions, such as a lack of political will, overreliance on external funding sources, limited access to enhancing capacity in advanced methods, and challenges analysing small sample sizes, which are typical in many studies in drylands, can impede the adoption of modern wildlife monitoring methods by local practitioners^30,46^. These factors, in turn, restrict local ability to use contemporary methods, such as AI for preprocessing datasets or hierarchical modelling for making robust inferences. Cumulatively, these challenges can result in information being generated but not utilised, as it takes too long to process or inappropriate metrics are used to infer abundance, for example. A collaborative network can address a small aspect of a much larger issue by providing analytical scripts and tutorials for managing datasets that comply with its metadata schema. These resources could address topics ranging from data manipulation to analysis and visualisation for reporting. Encouraging an inclusive network with membership across various organisations, including international and local universities and government agencies, can foster partnerships that support mentorship and collaborative programmes both domestically and internationally. Consequently, this can facilitate the adoption of modern methods, enhance local capacity, demonstrate the benefits of using contemporary techniques to other stakeholders and potentially help mitigate various socio-economic barriers.

### How would a network function at scale?

The growth of scientific communities operating at global and continental levels, along with the scientific papers generated by standardised networks of remote sensors, demonstrates that an initiative like a drylands collaborative network is achievable^42,47^. Establishing a network to bridge the gap between conservation activities and their outcomes through large-scale monitoring presents an opportunity to integrate often overlooked groups and employ innovative methods that combine data streams from different technologies, ultimately leading to more effective long-term outcomes. Any proposed network would need to scale from individual sites to a global level. One strategy is to utilise horizontal integration, whereby each data provider is responsible for their sampling, allowing flexibility in the survey design. However, by opting into the network and adhering to the established minimum standards, they will gain access to shared knowledge, a community of collaborators, and resources and tools in their native language to maximise their data and monitoring efforts. There are barriers to collating data from different sites, including ensuring that the datasets generated by various providers are interoperable. The transition from local sites to global analyses can only occur by providing clear guidance on the metadata schema. Research conducted under non-disclosure agreements can limit timely access to data and hinder broader knowledge sharing. Adopting frameworks such as Findable, Accessible, Interoperable and Reusable (FAIR)^48^, or following the approaches of globally successful initiatives like the Global Biodiversity Information Facility (GBIF)^49^, can facilitate the sharing of data once embargo periods have ended. For instance, data contributors could retain ownership while releasing datasets under permissive licences such as CC-BY following agreed embargo periods, or restrict access to approved network members during sensitive phases, as adopted by platforms such as GBIF and Wildlife Insights. Finally, by offering workshops and training events and inviting practitioners to contribute to the minimum monitoring standards, it ensures that any offerings align with community needs and are applicable, increasing the likelihood of wide adoption. By promoting widespread adoption, a network is more likely to attract funding bodies to become involved, which could alleviate some of the financial challenges of conducting long-term monitoring for its members, facilitate workshops, and ensure the network’s longevity. We demonstrate how a network could function at the scale of an individual site and a whole country using two case studies outlined below.

### Case study 1: local scale—NEOM Saudi Arabia

NEOM Nature Reserve is a newly-established protected area covering 25,000 km^2^ in northwest Saudi Arabia^50^. As part of the Kingdom’s Vision 2030, several larger natural landscapes are being protected and restored as sources for ecosystem restoration across the broader region. Within these areas, management interventions include regreening and wadi restoration, reducing human perturbation, and reintroducing ecologically functional communities of herbivores and carnivores. Bajdah Wildlife Reserve is one of the first ecological restoration sites established within NEOM Nature Reserve. It is currently an 85 km² fenced area, but plans are being established to expand it into a final designated reserve of 1086 km². In 2022, the reserve management facilitated the translocation and reintroduction of several native species, including sand gazelle (*Gazella marica*), Arabian gazelle (*Gazella arabica*), Arabian oryx (*Oryx leucoryx*) and Nubian ibex (*Capra nubiana*).

### Monitoring methods

**Fig. 2:**
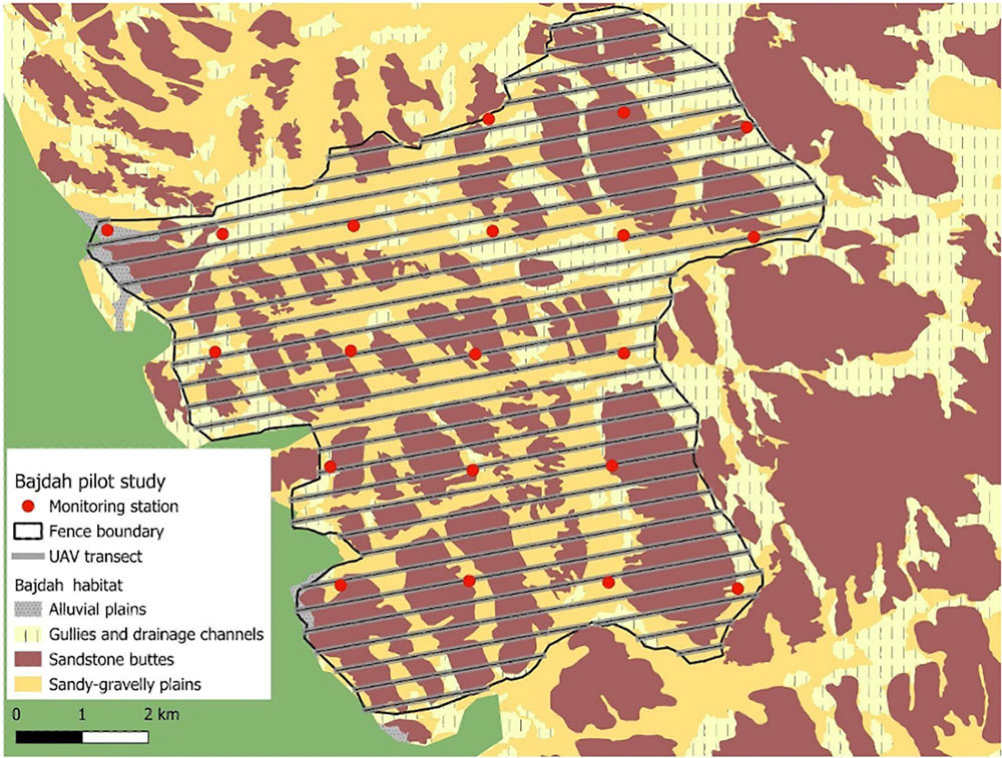
Locations of 20 systematically deployed monitoring stations, vegetation survey locations, and systematic UAV transects within Bajdah Wildlife Reserve.

NEOM land management teams with international collaborative support, including several of the co-authors of this paper, have implemented standardised protocols for camera traps, PAMs, UAVs, GPS telemetry and vegetation surveys in Bajdah Wildlife Reserve (Fig. 2). We monitored wildlife, including birds, and documented anthropogenic threats using 20 stations equipped with Browning StrikeForce camera traps and Audiomoth PAMs, placed at regular intervals of two kilometres within the protected area. Prior to release, we fitted GPS telemetry collars to three oryx, three Nubian ibex, three Arabian gazelle, and four sand gazelles, allowing reserve managers to monitor the status of a subset of the populations and quickly assess their distribution and movement within the reserve. We conducted UAV flights using a JOUAV CW-15 with a downward-oriented camera along 40 transects of varying lengths oriented from west to east to conduct strip counts for ungulates and measure vegetation structure within the reserve. We conducted vegetation surveys based on the rangeland condition score^51^ at each of the 20 monitoring sites. We classified the habitat surrounding the monitoring stations into four categories, based on measures of vegetation community composition, structure, and evidence of wildlife and disturbance. We also downloaded daily weather data from a remote weather station at the office in Bajdah Wildlife Reserve.

### Data processing and proposed analyses

**Fig. 3:**
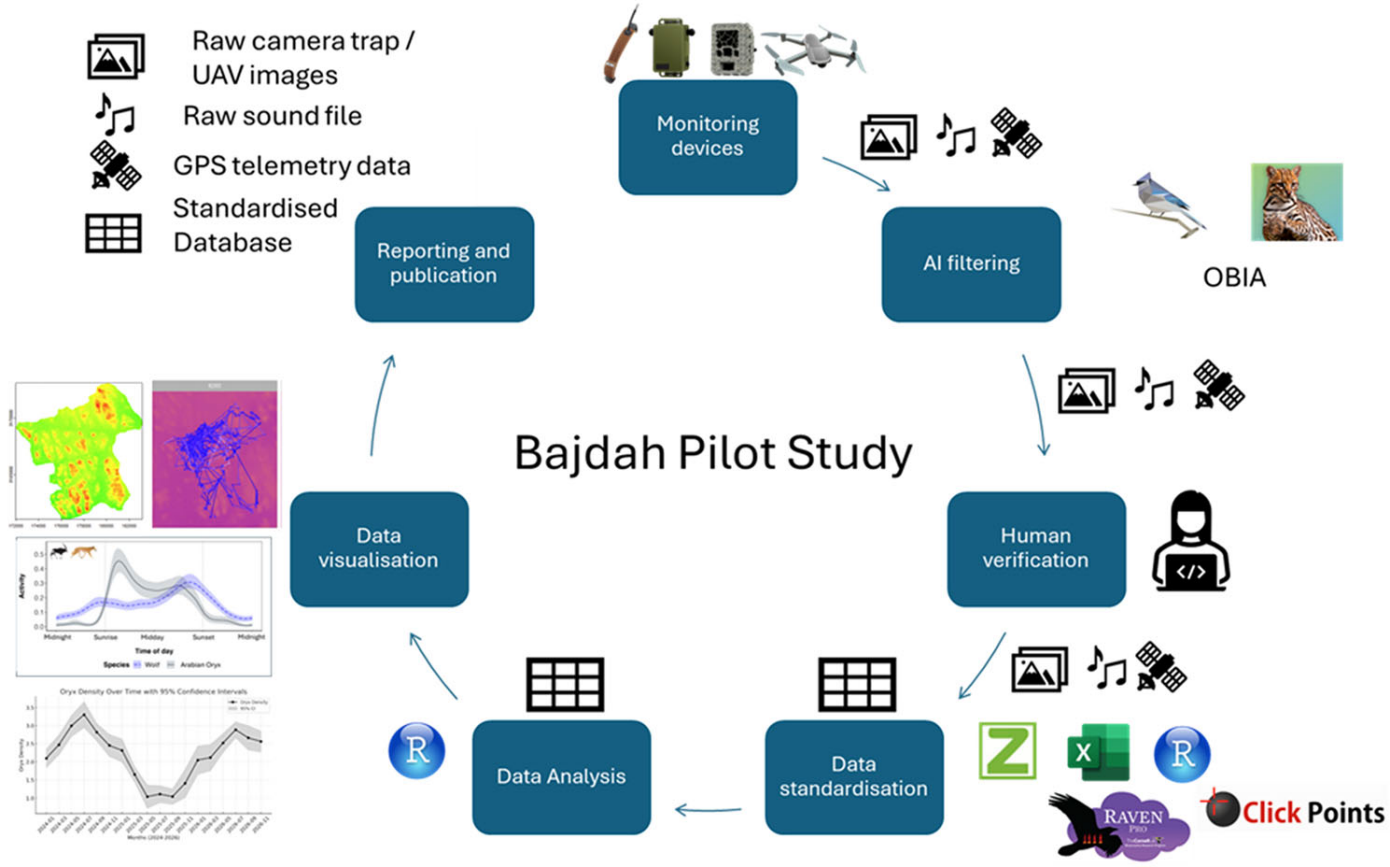
Diagram of the data cycle during the Bajdah Wildlife Reserve pilot study. Monitoring devices, including camera traps, PAM, UAVs, and satellite collars, are deployed in the field. Images and acoustic files are then processed through an AI filter (BirdNET, Megadetector, or other Object-based image analysis (OBIA)) before being verified by human experts. Verified images and sound files are converted into standardised Excel sheet formats in Zoological Society of London Camera Trap Analysis Package, ClickPoints, R Studio and Ravenpro, and then merged into the overall database. The dataset is then analysed in R Studio using the relevant packages (Table 1). Datasets are plotted onto appropriate visualisations, and tables summarising key statistics are prepared for export, reporting and publication in the final step.

We designed and implemented the monitoring stations, transect surveys, and regular ranger patrols to generate various metrics directly related to the objectives of reserve managers. Using the pilot study protocol, we anticipate that, at a maximum, if all devices are full, 5.12 TB of acoustic data, 600,000 camera trap images (1.36 TB), and 40 transects (114 GB) will be gathered monthly. We created the pilot study to test a workflow combining multiple data streams, while also developing initial code and modelling approaches at the scale of a single site. We followed the workflow illustrated in Fig. 3. We used AI to preprocess the camera trap, UAV images, and acoustic files, ensuring that human observers scrutinise only the files containing objects. We identified species from the camera trap and UAV images, with the distance from the camera trap measured by digitising the camera trap image to determine the animals’ position relative to the camera^52^. Acoustic data requires species identification but cannot be triangulated for distance sampling.

**Fig. 4:**
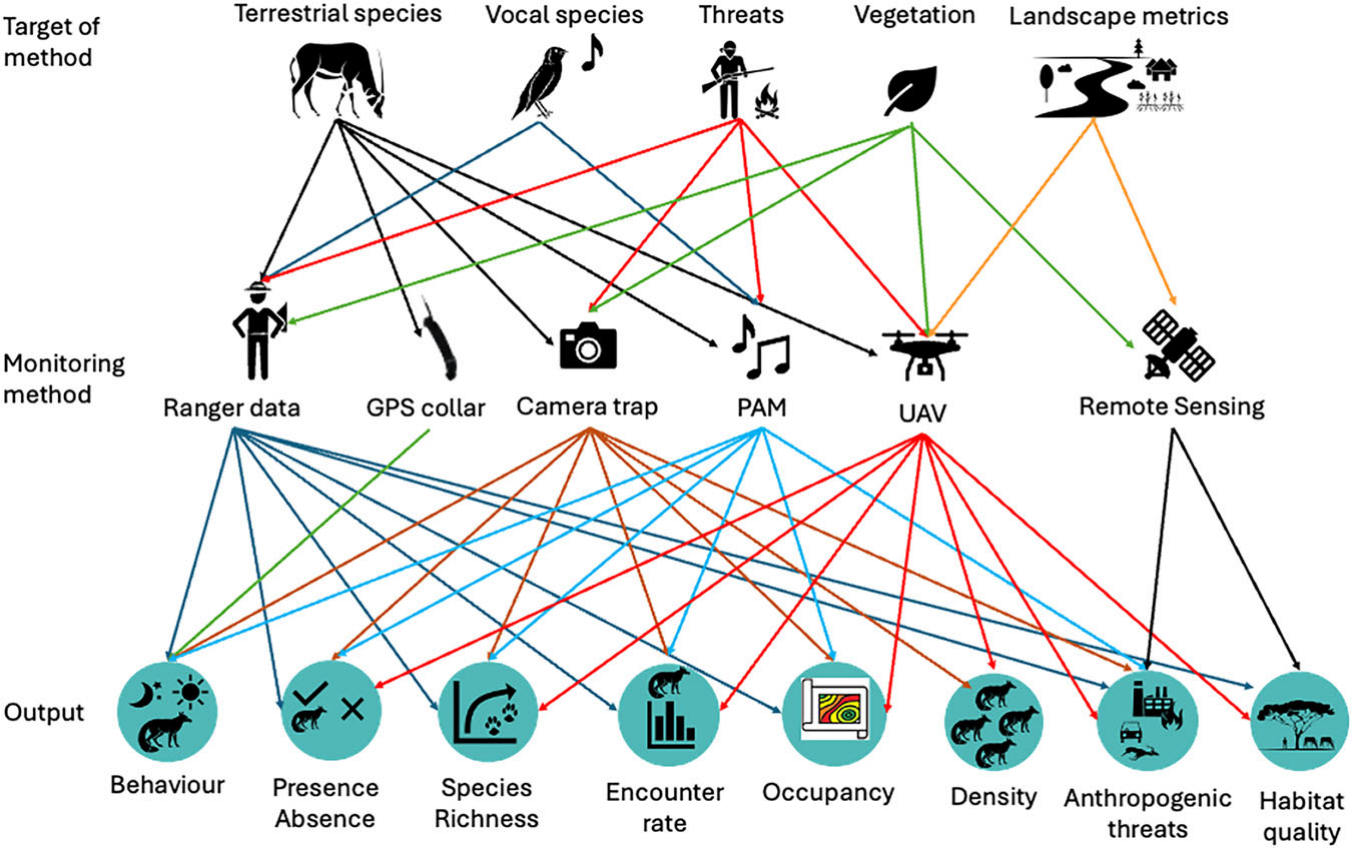
Overview of monitoring system arrays and approaches in a dryland network, illustrating the target species, the methods employed and the potential outputs and metrics derived from the data.

The Bajdah pilot study offers a unique opportunity to measure the efficacy of various remote sensing tools, given that the number of larger mammals in a semi-natural system is known. Thus, we are able to assess different methods and, by employing simulations informed by the data, provide recommendations for a potentially scalable and dependable monitoring protocol. Figure 4 summarises how each sensor type could contribute to specific conservation or monitoring goals in drylands generally. We aimed to produce the following outputs from the pilot study in Bajdah, with the datasets used to address each output in parentheses:

1. An assessment of vegetation condition scores at each of the 20 monitoring stations in Bajdah Wildlife Reserve (Vegetation survey).
2. Interpolated heatmaps of anthropogenic threats, highlighting their intensity and distribution within the reserve (Camera trap, PAM and UAV).
3. Occupancy models will be used to identify the biotic and abiotic factors that most accurately describe the distribution of key species within the Bajdah Wildlife Reserve (Camera trap and PAM).
4. Density estimates for all the reintroduced ungulate species, including their precision and required effort for each method. These will be compared to the number released into the reserve to determine the most appropriate method or combination of methods for large-scale monitoring (for example, camera traps, UAVs and Ranger patrols).
5. Species accumulation curves for mammals and birds help understand how many species have been detected and whether we require more survey effort to derive a more complete species list (Camera trap and PAM).
6. Behavioural analyses of reintroduced species, including circadian activity patterns, home range and distribution within the reserve (Camera trap, UAV and GPS telemetry).

### Case study 2: large-scale networks and longevity—The Wildlife Observatory of Australia (WildObs)

Some barriers to establishing large-scale collaborative networks include attracting long-term investment from funding agencies and encouraging disparate groups to collaborate (such as academics, non-government and government organisations). The Wildlife Observatory of Australia (WildObs) was established in 2023 to provide a home for all types of camera-trap users to develop and share tools, knowledge, and data. It provides users with cloud-based image storage and AI-powered species identification, standardised spreadsheets for personal analyses in the camtrapDP format^53^ and automated reports, including multi-site, multi-season hierarchical detection-corrected occupancy and abundance analyses^54^.

WildObs was funded through a co-design process with the Australian Research Data Commons (ARDC)^55^ to develop shared infrastructure as part of the Planet program’s ‘machine-based observations’ initiative. WildObs partnered with Australia’s other biodiversity data organisation, such as the Terrestrial Ecosystem Research Network (TERN), to publish presence-absence survey datasets and contribute to the Threatened Species Index (TSX) and the Atlas of Living Australia (ALA), to publish curated sets of tagged images for training species classifiers. WildObs primary goal is to serve as the repository of Australian camera-trap data for government purposes, which is crucial for tracking threatened species. However, WildObs stakeholders and users span many sectors, which poses some challenges as these groups have different objectives, ranging from management (e.g. lethal control of invasive species) to research, to supporting environmental markets where there is potential to sell biodiversity credits. Therefore, WildObs’ steering committee has diverse stakeholders representing these varied use cases.

### Establishing a network

**Fig. 5:**
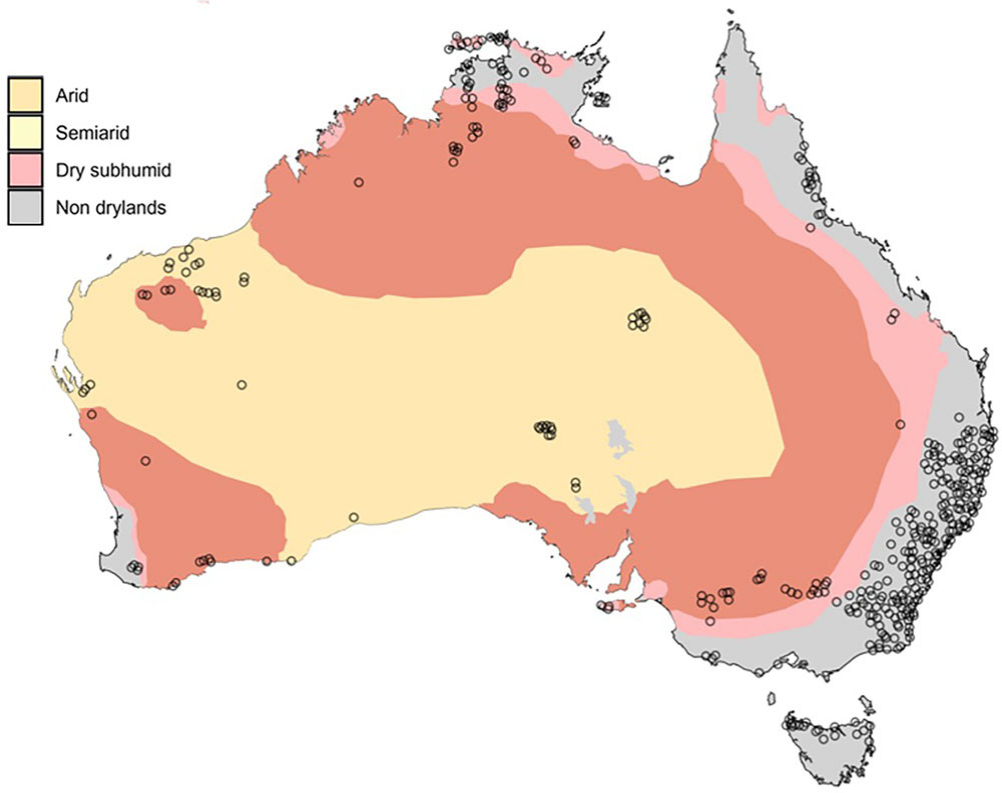
Camera locations from all WildObs providers were grouped into individual landscapes by buffering each camera by 5 km and merging overlapping areas. Each distinct landscape, represented by a hollow black point, illustrates the spatial distribution of camera traps across the WildObs network. Dryland habitat data from: UNEP-WCMC^109^.

A dryland network could emulate this community-led approach to clearly identify the community’s appetite for such a project and its specific needs, thereby bolstering support from the outset. To this end, WildObs has demonstrated practical concepts useful for any network development. To identify the needs of the wildlife monitoring community in Australia, WildObs hosted a ‘Wildlife Summit’ with 30 leaders representing various disciplines and organisations such as the Bush Heritage and World Wildlife Fund (WWF), multiple levels of state government (Queensland Department of Technology, Science and Innovation; Queensland Parks and Wildlife Service), federal government representatives (DCCEW), and several universities^7^. The Wildlife Summit revealed an appetite for shared infrastructure, knowledge sharing, data sharing and collaboration. Next, to identify specific camera usage and user needs, they conducted a literature review and a questionnaire of over 150 camera users, which revealed that while small targeted camera trap surveys were prevalent across the continent, there had been minimal expansion into multi-site, multi-year studies, partly due to limited human bandwidth and exposure to more efficient technologies^7^, such as AI-powered object detectors (e.g. MegaDetector) and computer vision classifiers for identifying species in images. There was also little use of detection-corrected hierarchical models to address slight variations in deployment methodology (e.g. occupancy models with covariates in the detection formula), thereby enabling multiple studies to be integrated into a monitoring framework. There were also friction points to collaboration required for a large-scale, long-term wildlife monitoring community in Australia. At the time of writing, WildObs has >100 different data contributors across all Australian states (Fig. 5). To ensure datasets are interoperable, there is a rigorous standardisation pipeline that includes sending providers detailed metadata questionnaires. The final output complies with camtrapDP’s metadata schema, making it suitable for other initiatives and meeting WildObs’ requirements^53^.

As the use of wildlife cameras increases, WildObs considered scaling issues from the outset. This underpinned WildObs’ decision to build and host everything on federal digital infrastructure (the Nectar Research Cloud) to enable cloud storage and computing. For a dryland observatory spanning multiple countries, collaboration with a global provider such as Google, Microsoft or Amazon, similar to the Wildlife Insights platform, could be a viable alternative. However, this would need to be weighed against any prospective data-sharing agreements and privacy concerns.

### Analysing network data

Most networks have users contributing data from different habitats, containing different species and sometimes using different sampling methods, all of which was true for camera usage in Australia. Analysis of such messy datasets requires more careful data preparation and nuanced statistics that explicitly include these sources of variation. An example of how WildObs accomplished this was for a review of faunal impacts following the Australian black summer bushfires of 2019–2020, which serves as a poignant example of climate change-induced extreme weather events. The fires burned over 10 million hectares and potentially impacted 3 billion animals^56,57^. A consortium of NGOs established the Eyes on Recovery project to assess faunal impacts using cameras, deploying 1100 cameras across 17 different surveys in 9 landscapes, which produced 8.5 million images^58^. This exemplifies a distributed network, as each of the 17 surveys was led by a local partner who deployed cameras in various ways to target different taxa and address their management questions. WildObs standardised and analysed data from 13 of the 17 camera deployments, each targeting different taxa, by using the Google Earth Engine Burnt Area Map remote sensing layer for fire severity and other environmental covariates as explanatory variables^59,60^. Hierarchical occupancy models were employed to account for the effect of different camera deployment strategies on wildlife detectability. This was achieved by assessing whether variables related to camera deployments, such as camera brand, whether the camera was positioned on a road or a trail, or whether a lure was used, improved the model’s fit before estimating occupancy for each species^54^. When analysing the data across large spatial and temporal scales, no consistent effect of fire on Australian fauna was detected^58,59^. This example illustrates how a horizontal network operates, with individual practitioners responsible for their fieldwork, uploading images and metadata, and verifying AI-powered species identification. Meanwhile, WildObs led the data curation, standardisation, analysis, and report writing. At the time of the project (2023), the code base was still being developed, and the WildObs portion of the project took 10 weeks, while today, the same project has a 2-week turnaround time. This demonstrates that WildObs’ approach can go from images to inferences within time periods relevant to management.

## Conclusion

A collaborative network focused on dryland research could yield important insights into ecosystem changes at various scales, evaluate the effectiveness of interventions such as rewilding, and quantify ongoing and emerging threats to dryland biodiversity. The network must rely on a coalition of practitioners, experts and relevant capacity-building programmes. It must develop multi-scale methodological frameworks that function effectively for both small protected areas and extensive dryland regions, including those spanning multiple nations. Valuable lessons can be learnt from other large-scale initiatives, such as WildObs in Australia, to understand how to effectively build multi-partner networks that ensure sufficient engagement and scalability.

Many regional programmes, such as Saudi Vision 2030, are supported by targets and aims similar to those of the Kunming-Montreal Global Biodiversity Framework’s (KM-GBF) 30 by 30 targets or the United Nations Sustainable Development Goals (SDG)^61,62^. A network would enable its members to generate metrics to demonstrate whether targets like SDG 15 ‘Life on Land’ are being achieved by monitoring trends in terrestrial ecosystems, the species within them, and the integrity of their habitats. There are funds linked to aligning with global biodiversity initiatives. For example, the Global Biodiversity Framework Fund supports projects aiming to meet the KM-GBF targets, emphasising national-level biodiversity governance, and as of June 2025, it had $386 million pledged^63^. By aligning with global biodiversity frameworks, dryland practitioners can utilise an accountable, transparent approach that helps secure long-term funding and promotes sustainable resource use and engagement with local communities.

Collectively, any potential network must develop and enhance the analytical tools necessary to fully leverage data, particularly when integrating information from different species or dryland study sites and employing diverse data collection methods. Dryland practitioners require a formal understanding of which data is most reliable for specific questions and the best ways to combine different data types. Unlocking the full potential of data (e.g. sensor data) involves addressing the varied field methods, which differ by location, season, study duration, and design. To tackle this, it is necessary to focus on exploring, testing, and developing analytical tools. The use of pilot studies conducted in semi-natural conditions, such as fenced areas like Bajdah Wildlife Reserve, will facilitate the evaluation and comparison of different methodologies and analytical tools. However, for these approaches to be viable, they must be applicable beyond fenced areas if rewilding is to take place on a large scale and fulfil the underlying ambitions of those implementing it. This will ultimately provide guidance on how to align diverse data with appropriate statistical frameworks, enabling accurate inferences about dryland wildlife (i.e. ecological communities composed of multiple species), the environment, and their responses to changes at scale.

Currently, one of the main barriers to collaboration is that metadata describes study design, and deployments are not interoperable. Before establishing a network, dryland researchers already using any of these monitoring methods should strive to adopt rigorous and widely recognised metadata schemas, such as camtrapDP, for their ongoing work^53^. Ground-truthing outputs from new technology or methods, such as land use classification products derived from remote sensing imagery^64^, would also benefit a global network by reducing delays caused by waiting for these steps to be completed. Initial steps for a network could include appointing a steering committee to set clear goals and communicate mission statements to the community. The committee should identify key stakeholders to gain insight into community needs, historical barriers to collaboration, and which data-sharing approaches resonate most with the community, such as WildObs, and facilitate pilot studies with multiple data streams from different regions, similar to Bajdah Wildlife Reserve. This will help understand how the network can scale as engagement increases.

## Data Availability

Camera-trap study locations were obtained from Wildlife Insights (https://www.wildlifeinsights.org). Passive acoustic monitoring (PAM) study locations were obtained from the Worldwide Soundscape database (https://zenodo.org/records/14216871). Animal tracking (GPS) study locations were obtained from Movebank (https://www.movebank.org) and accessed via the platform’s recommended API. Dryland habitat data were obtained from the UNEP-WCMC Dryland Habitat Dataset (https://data-gis.unep-wcmc.org/portal/home/item.html?id=789fcac8959943ab9ed7a225e5316f08). Study locations for Fig. 5 are available upon request from the Wildlife Observatory of Australia (https://wildobs.org.au/). Colour symbols for camera trap, PAM and GPS collar were used courtesy of the NESP Resilient Landscapes Hub (https://nesplandscapes.edu.au). Black silhouette images in Fig. 4 were created using a combination of symbols courtesy of the Spatial Monitoring and Reporting Tool (SMART; https://smartconservationtools.org) and PowerPoint.
